# X-linked inheritances recessive of congenital nystagmus and autosomal dominant inheritances of congenital cataracts coexist in a Chinese family: a case report and literature review

**DOI:** 10.1186/s12881-019-0780-4

**Published:** 2019-03-19

**Authors:** Naihong Yan, Lirong Xiao, Chen Hou, Bo Guo, Wei Fan, Yingping Deng, Ke Ma

**Affiliations:** 1 Research Laboratory of Ophthalmology and Vision Sciences, Torsten-Wiesel Research Institute of World Eye Organization, West China Hospital, Sichuan University, Chengdu, 610041 China; 2 Department of Ophthalmology, West China Hospital, Sichuan University, Chengdu, 610041 China

**Keywords:** Case report, Congenital nystagmus, Congenital cataracts, FRMD7, GJA8, Chinese pedigree

## Abstract

**Background:**

Congenital nystagmus (CN) and congenital cataracts are distinct eye diseases and are usually isolated. Cases with CN and congenital cataracts caused by different genes in one family have been rarely reported.

**Case presentation:**

A 27-year-old man presented with CN and congenital cataracts and he underwent cataract extraction 2 weeks after birth. Three years later, he had posterior chamber intraocular lens implantation. The proband’s mother was only afflicted by bilateral lens opacities. Lensectomy was performed in both eyes at age 15. The proband’s daughter had bilateral central cataracts and no nystagmus. She had undergone cataract extraction when she was two months old. In this family, 8 affected individuals were affected by bilateral cataracts, and three of them presented with CN. The genetic analysis was performed using a specific Hereditary Ophthalmological Disease Gene Panel on proband and his parents (one of which was a patient). PCR and Sanger sequencing verified the presence of these variants in all members of the family. The novel mutation, c.498-3C > T, in *FRMD7* explains why X-Linked recessive inheritance of CN was found in a subset of patients. A heterozygous mutation of the *GJA8* gene (c.139G > C), was identified in all patients and thus explains the autosomal dominant pattern of inheritance of congenital cataracts within the family.

**Conclusions:**

This is the first time that *FRMD7* and *GJA8* gene mutations have been linked to the pathogenesis of a family with both CN and congenital cataracts. The phenomenon of two different genetic patterns coexisting in one family is rare.

**Electronic supplementary material:**

The online version of this article (10.1186/s12881-019-0780-4) contains supplementary material, which is available to authorized users.

## Background

Congenital nystagmus (CN) are ocular motor disorders in which patients are afflicted by periodic involuntary ocular oscillations affecting both eyes [1, 2]. Disease onset normally occurs at birth or develops shortly thereafter. The inheritance model of CN has been previously described in various forms as being either autosomal or X-linked, and either dominant or recessive, with X-linked inheritance and incomplete penetrance being the most common [3]. Three distinct X-linked loci are known: Xp11.4-p11.3, Xq26-Xq27, and Xp22.3-p22.2 [4–6]. The Xq26-q27 and Xp22.3-p22.2 regions contain genes coding for FERM domain-containing 7 (*FRMD7*) and G-protein coupled receptor 143 (*GPR143*), respectively, and both of these genes have been identified as contributors to CN disease [6, 7]. The *GPR143* gene is also associated with X-linked ocular albinism type 1 (OA1) [8, 9].

Congenital cataracts are by far the most common explanation for blindness in children globally, with such blindness being characterized by lens opacity [10]. It is estimated that blindness occurs in approximately 1–6 of every 10,000 births in highly developed countries, and at higher rates of 5–15 per 10,000 births in those countries which are poorer [11–13]. As many as one in three congenital cataracts are believed to be linked to specific genetic mutations [14, 15]. Over 48 genes have been identified in the inherited forms of isolated or primary cataracts with minimal other ocular signs [15]. Most often, inherited cataracts not associated with another known disease present a pattern of autosomal dominant (AD) inheritance, but this is not always the case and in some instances X-linked or autosomal recessive (AR) versions are evident [16].

In our study, four generations of a family from China afflicted CN and congenital cataracts were recruited. Some of the affected individuals exhibited CN, and all were afflicted by congenital cataracts. Patients were sequenced to find candidate genes within the family. We identified two different genetic patterns that coexist in the family. Mutations in *FRMD7* and *GJA8* genes were responsible for the pathogenesis of CN and congenital cataracts respectively.

## Case presentation

**Fig. 1:**
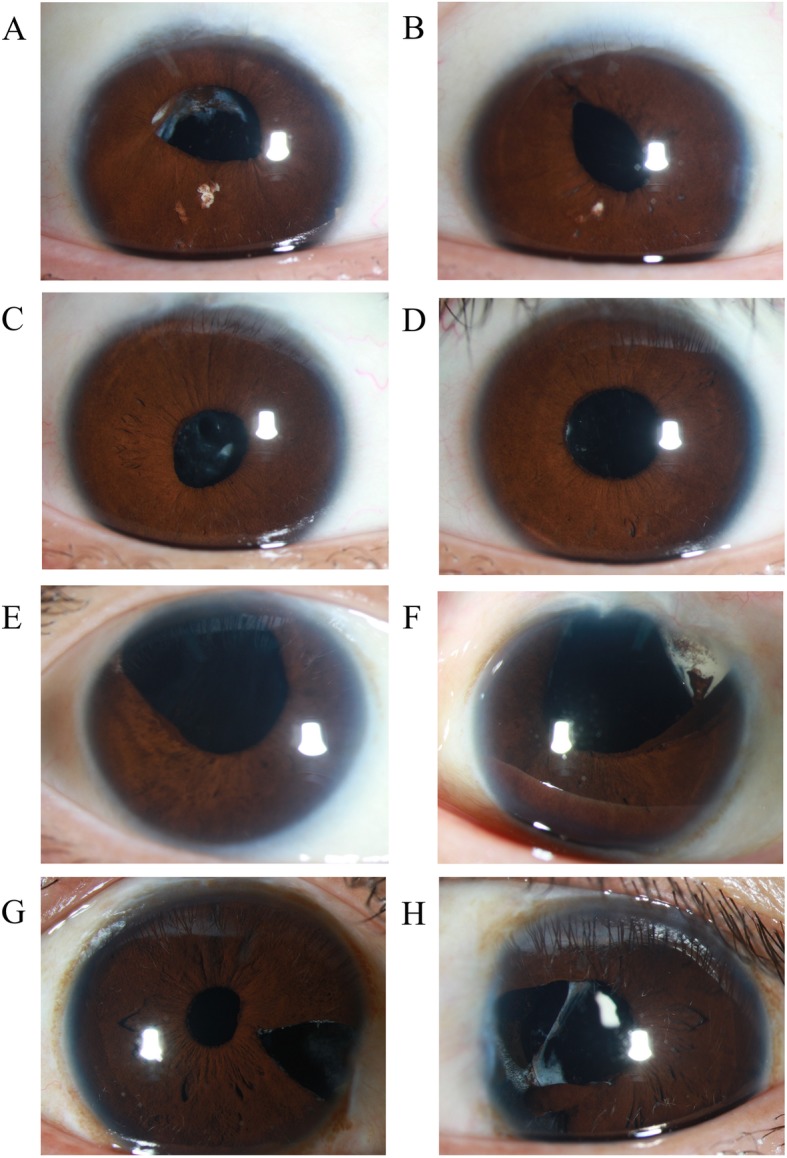
Slit-lamp photograph of patients who had congenital cataracts. **a**: Right eye of the proband III:1. The pupil is upward. Thickened capsule can be seen. Intraocular lens is located in the right position. **b**: Left eye of the proband III:1. The pupil is not round. Intraocular lens is located in the right position. **c**: Right eye of patient III: 3. Pupil is not perfectly round. **d**: Left eye of patient III:3. Pupil is round. Intraocular lens is located in the right position. **e**: Right eye of patient II:1. Irregularly shaped pupil can be seen. Aphakia. **f**: Left eye of patient II:1. The iris has anterior adhesion from the 3 o’clock to 5 o’clock position. **g**: Right eye of patient II:3. There is a hole of circumferential iridectomy. Intraocular lens is located in the right position. **h**: Left eye of patient II:3. The pupil deformation is severe with capsule thickened

**Fig. 2:**
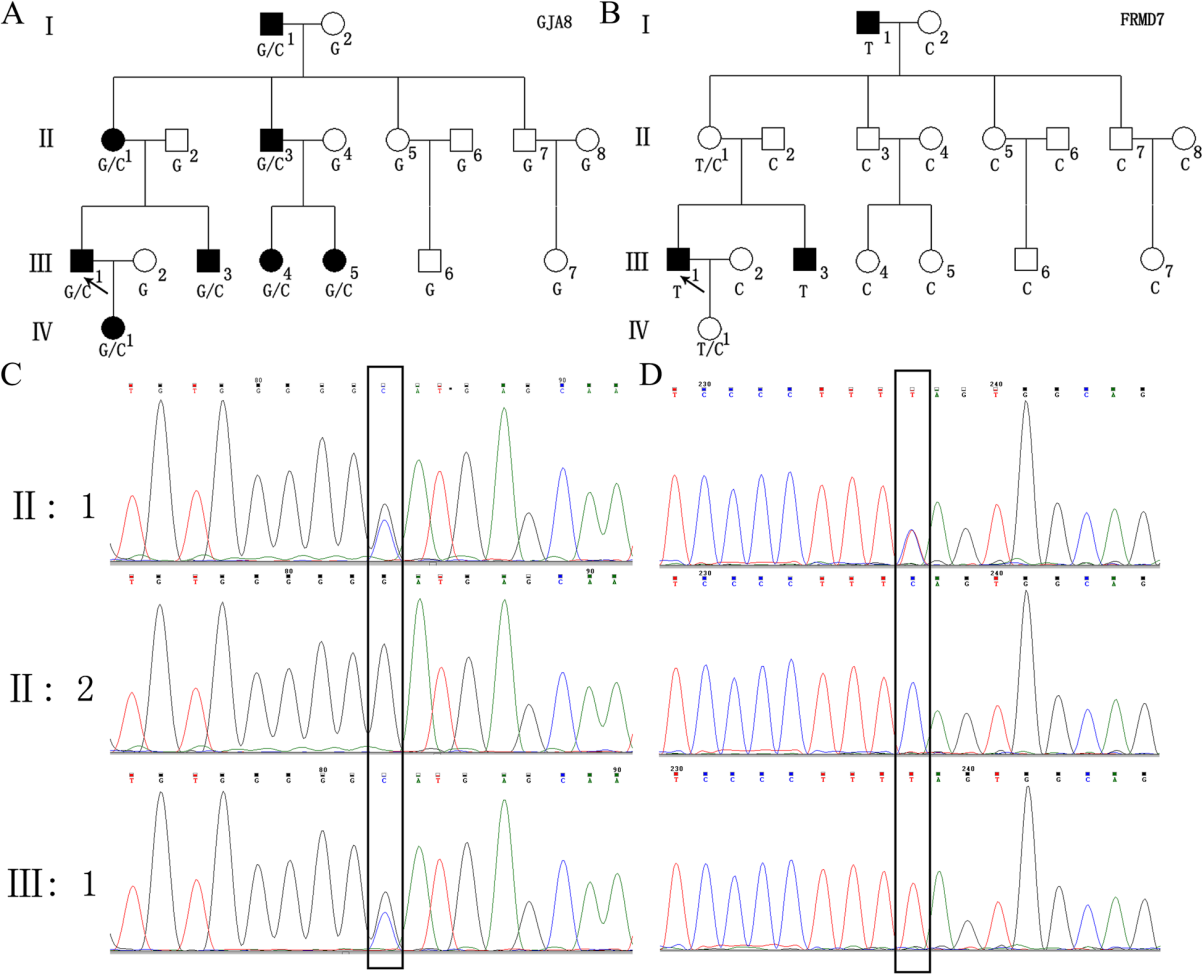
*GJA8* and *FRMD7* mutations in this family. **a** and **c** The *GJA8* heterozygous mutation c.139G > C was found in all patients and likely is responsible for the autosomal dominant pattern of inheritance of congenital cataracts in this family. **b** and **d** The *FRMD7* splicing variant c.498-3C > T was found in I:1, III:1 and III:3; thus, this variant likely plays a role in CN’s X-Linked recessive inheritance

The proband (patient III: 1, Fig. 1a, Fig. 1b, Fig. 2a) is a 27-year-old who previously underwent cataract extraction 2 weeks after birth. Three years later, he had posterior chamber intraocular lens implantation but he did not receive any amblyopia treatment, nor did he use aphakic spectacle for visual rehabilitation following the two surgeries. He was found to have nystagmus on the fortieth day after birth and was diagnosed with CN. His daughter (IV: 1) had bilateral central cataracts and no nystagmus. She had undergone cataract extraction when she was two months old. Visual rehabilitation via aphakic spectacle correction using + 20 diopter sphere (DS) in the right eye and + 21DS in the left eye was performed.

The proband’s brother (III: 3, Fig. 1c, Fig. 1d) had bilateral cataracts and conjugate horizontal nystagmus. He underwent cataracts extracted at age 6 and had an intraocular lens implanted at age 11. The proband’s mother (II:1, Fig. 1e, Fig. 1f) was additionally afflicted by bilateral lens opacities. Lensectomy was performed in both eyes at age 15. The proband’s uncle (II:3, Fig. 1g, Fig. 1h) also had bilateral congenital cataracts without nystagmus. He had phacoemulsification cataract extraction and intraocular lens implantation when he was 28 years old. His two daughters (III: 4, III: 5) were found to have bilateral central cataracts without nystagmus. They both had phacoemulsification cataract extractions and intraocular lens implantations when they were 9 years old. The patient features are described in Table 1 and this family was recruited from West China Hospital, Sichuan University. All participants were informed about the purpose of the protocol and signed consent forms. The protocol was approved by the Ethics Committee of West China Hospital, Sichuan University.

**Table 1:** Summary of clinical features of patients

ID	Gender	Age (years)	Congenital nystagmus	Congenital cataracts	Cataract surgery	BCVA (OD/OS)
I: 1	Male	74	Yes	Yes	No	HM/HM
II: 1	Female	48	No	Yes	15 years old	HM/HM
II: 3	Male	45	No	Yes	28 years old	0.2/0.06
III: 1	Male	27	Yes	Yes	cataract extraction 2 weeks after birth, lens implantation at 3 years old	0.02/0.2
III: 3	Male	24	Yes	Yes	cataract extraction at 6 years old, lens implantation at 11 years old	0.02/0.1
III: 4	Female	20	No	Yes	9 years old	0.1/0.08
III: 5	Female	21	No	Yes	9 years old	0.2/0.1
IV: 1	Female	1	No	Yes	cataract extraction two months after birth	−/−

Patient III:1, his mother (II:1, patient) and his father (II:1, normal) were sequenced by with a specific Hereditary Ophthalmological Disease Gene Panel. DNA was extracted using QIAamp DNA blood mini kit (Qiagen) and exons coinciding with genes of interest being captured via the Panel with biotinylated oligo-probes (GenCap Enrichment Technologies, MyGenostics, Beijing). A total of 662 genes, including most known to related to hereditary ophthalmological disease, were included in this panel (see Additional file 1: Table S1). An Illumina Solexa HiSeq 2000 sequencer (MyGenostics, Beijing) was used for sample sequencing. Bioinformatics analysis was performed to identify the mutations were linked to the disease phenotype present in the affected family. Sanger sequencing was verified the variants in other individuals using primers: *FRMD7* (NG_012347) forward primer CATCTGGCACAAACTCGGTA and reverse primer CTCTTAAAACTCAACTTGCGGA. *GJA8* (NG_016242) forward primer GAACATCTTGGAGGAGGTGAAT and reverse primer CAGAGGCGAATGTGGGAGAT.

More than 99% of the targeted regions were covered in each sample. Using bioinformatics analysis, two candidate mutations were identified in this family. A heterozygous mutation in *GJA8* gene (chr1–14,738,022, exon2, c.139G > C, p.D47H, NM_005267.4) and a novel *FRMD7* gene splicing mutation (chrX-131,219,759, exon7, c.498-3C > T, splicing, NM_194277.2) were found in patient III:1 (Other variants results of Patient III:1 to see Additional file 2: Table S2). The c.139G > C mutation of *GJA8* gene was found in ClinVar database (https://www.ncbi.nlm.nih.gov/clinvar/variation/280147/) (Clinical significance: Pathogenic) and not found in gnomAD database. The c.498-3C > T mutation of *FRMD7* gene was not found in ClinVar database and gnomAD database. Segregation analysis was performed in the other family members using Sanger sequencing. The *GJA8* heterozygous mutation c.139G > C was found in all patients and is likely responsible for autosomal dominant inheritance of congenital cataracts (Fig. 2a, Fig. 2c). The *FRMD7* splicing variant c.498-3C > T was found in I:1, III:1 and III:3. II:1 and IV:1 were carriers (Fig. 2b, Fig. 2d). This segregation pattern is consistent with X-Linked recessive inheritance. These two mutations had paternal origin and came down from I:1, and the mutations were absent in those family members unaffected by disease. The sequence results of all the patients and some normal family members were shown in the Additional file 3: Figure S1 and Additional file 4: Figure S2.

A computational analysis of the D47H *GJA8* mutant using a Polymorphism Phenotyping (PolyPhen-2) analysis yielded a result predicting this mutation to be “probably damaging”, while Sorting Intolerant From Tolerant (SIFT) analysis similarly suggested an intolerant substitution. Human *FRMD7* is 2145 bp in length, with a total of 12 exons. A novel splice variant c.498-3C > T of *FRMD7* had been found comparing with the original form of *FRMD7.* A novel isoform of *FRMD7* arises through the alternative splicing of *FRMD7* mRNA, leading to the deletion of 148 bp in exon 4. Through the “Deep Learning” algorithm of SPIDEX, the dpsi_max_tissue score was − 0.1228, and the dpsi_z score was − 0.514. The score range is − 100 to 100. The closer the absolute value of the score is to 100, the greater the influence of mRNA splicing. The dbscSNV analysis found that the ada_score was 0.6943564 (the score range is 0–1, the greater the score is, the greater the impact; the normal value is no more than 0.6), and the rf_score was 0.232 (the score range is 0–1; the greater the score is, the greater the impact; the normal value is no more than 0.6). If one of these scores is greater than 0.6, dbscSNV is T (TRUE), and otherwise, it is F (FALSE) (Table 2). Accordingto the ACMG guidelines, the c.139G > C variation of *GJA8* gene was “pathogenic” and the c.498-3C > T variation of *FRMD7* gene was “likely pathogenic”.

**Table 2:** In Silico Prediction of c.139G > C of *GJA8* and c.498-3C > T of *FRMD7*

PolyPhen	score	meaning
	1.000	probably damaging
SIFT	score	meaning
	0.02	intolerant
SPIDEX:	dpsi_max_tissue	dpsi_zscore
	−0.1228	−0.514
dbscSNV:T(TRUE)	ada_score	rf_score
	0.6943564	0.232

## Discussion and conclusions

A Chinese family affected both by CN and by congenital cataracts was reported in our study. The phenomenon of two different types of eye diseases with different genetic patterns of inheritance in a family is very rare. No similar results have been reported.

The D47H *GJA8* mutation has previously been linked to congenital nuclear and zonular pulverulent cataracts, and has the same cataract type as this family [17]. The *GJA8* coding region consists of one exon and encodes 432 amino acids. Over 24 distinct *GJA8* mutations have been reported to date in humans and in mouse models, with direct evidence that these mutations promote the formation of cataracts [18]. The c.139G > C substitution leads to the substitution of a histidine in place of aspartic acid at position 47, leading to a change from negative to positive charge [17]. Aspartic acid at position 47 is found in the extracellular loop E1 region of *GJA8* [19]*.* Consistent with Li’s study, our PolyPhen and SIFT results suggest that D47H is a likely loss-of-function mutation [17].

It has been reported that the knockout of *GJA8* in mice results in cataract development the impairment of lens growth [20]. *GJA8* is highly expressed in both epithelial and lens fiber cells, particularly during their differentiation [21]. The mutated *GJA8* alters lens fiber cell formation, which in turn leads to cataract formation [20].

*FRMD7* mutations are major causes of CN [7]. *FRMD7* expression is primarily detectable within the retina and vestibular system, with additional expression in portions of the brain regulating the vestibulo-ocular reflex [7, 22]. It has been reported that *FRMD7* is important to facilitate neuronal circuit asymmetry for directional selectivity [23]. Nevertheless, exactly what role is played by FRMD7 is still uncertain. The protein encoded by *FRMD7* has an N-terminal FERM domain that may facilitate signal transduction, similar to other proteins in this family with this same domain [23].

Interestingly, most mutations leading to congenital nystagmus are located in this FERM domain [22].

A *FRMD7* splice variant (*FRMD7-S*) has previously been cloned and identified. This variant form may be important in the context of neuronal differentiation and development [24]. Another splice variant, *FRMD7* (*FRMD7_SV2*), is similarly predicted to be important for neuron development [25]. The *FRMD7* mutation of c. 206-5 T > A is predicted to disrupt the splice acceptor site in the third intron, while variant c.205 + 2 T > G is predicted to be pathological on the basis of its likelihood to induce nonsense-mediated decay or exon skipping [26]. In this family, a novel splice variant of *FRMD7,* c.498-3C > T, has been identified. This splice variant was predicted to be harmful using bioinformatics analysis and this variant is likely the causative lesion for CN in this family.

In summary, this study reveals two variants of two genes. These variants explain two clinical pathologies with different inheritance patterns in a Chinese family. The exact means by which these variants result in CN and congenital cataracts at the molecular level remains to be determined, and further functional studies will be necessary to offer novel insights into this inherited ocular disease.

## Additional files

Additional file 1: **Table S1.** The panel of genes screened for the family (662) (XLSX 17 kb)

Additional file 2: **Table S2.** Other variants results of Patient III:1. (XLSX 14 kb)

Additional file 3: **Figure S1.** Sanger sequence of *GJA8* gene. The sequence results of *GJA8* c.139G > C mutation in all the patients and some normal family members. (TIF 3118 kb)

Additional file 4: **Figure S2.** Sanger sequence of *FRMD7* gene. The sequence results of *FRMD7* c.498-3C > T splicing variant in all the patients and some normal family members. (TIF 2876 kb)

## Abbreviations

AD: autosomal dominant
AR: autosomal recessive
CN: Congenital nystagmus
DS: diopter sphere
FRMD7: FERM domain-containing 7
GPR143: G-protein coupled receptor 143
OA1: ocular albinism type 1
PolyPhen-2: Polymorphism Phenotyping
SIFT: Sorting Intolerant From Tolerant
